# Saved by Wealth? Income, Wealth, and Self-Perceived Health in Spain during the Financial Crisis

**DOI:** 10.3390/ijerph17197018

**Published:** 2020-09-25

**Authors:** Guillem López-Casasnovas, Marc Saez

**Affiliations:** 1 Center for Research in Health and Economics (CRES), Universitat Pompeu Fabra, 08005 Barcelona, Spain; guillem.lopez@upf.edu; 2 Department of Economics and Business, Universitat Pompeu Fabra, 08005 Barcelona, Spain; 3 Barcelona Graduate School (BGSE), Universitat Pompeu Fabra, 08005 Barcelona, Spain; 4 Research Group on Statistics, Econometrics and Health (GRECS), University of Girona, 17003 Girona, Spain; 5 CIBER of Epidemiology and Public Health (CIBERESP), 28029 Madrid, Spain

**Keywords:** self-assessed health, wealth, wealth composition

## Abstract

We evaluate the association between the variations in income and wealth, (both aggregate and split between real estate and financial wealth), and self-perceived health in Spain using a longitudinal sample of individuals before and after the financial crisis. We estimated generalized linear mixed models, with a binomial response and a logistic link, for four waves of the Spanish Survey of Household Finances (two before and two after the crisis), adjusting for variables at the family and individual levels. We also controlled for familial and individual heterogeneity and for temporal trends. While an increase in wealth greatly increases the probability of younger individuals reporting better health, this is not the case for older individuals. Decreases in gross wealth are associated with decreases in the probability of declaring good/very good health only in families whose reference person is over 44 years old. We conclude that: (i) not just income but net wealth effects impact on the consequences of income fluctuations on consumption and health assessed, (ii) the composition of individuals’ net wealth may also matter, since they are differently affected by the shocks in the economic crisis, (iii) age plays a significant role and, finally, (iv) individual reactions in terms of consumption and savings, given any level of income and wealth, according to the risk aversions for precautionary idiosyncratic motives, may also need to be considered in order to complete the picture.

## 1. Introduction

There is an extensive body of literature analyzing socioeconomic inequalities in well-being (for a review, see O’Donnell et al. [1]). A better socioeconomic position is generally associated with both higher average and lower variation in self-reported health. The earlier papers in this body of literature use level of education as surrogates of socioeconomic conditions. More recently, the availability of administrative data has allowed tax records to be used to measure socioeconomic conditions. 

However, the joint role wealth and income have in shaping well-being has been studied to a much lesser extent; especially for younger adults. Notable exceptions to this are the work of Poterba et al. in the case of retirement [2], Schwandt [3] and Pool et al. [4] for wealth shocks, Finkelstein et al. [5] for wealth, health, and well-being, Liu and Menegatti for wealth investment and health [6], and Blázquez and Budria [7] and Saez et al. [8] for population health in Spain during the financial crisis. Most of the literature, however, has focused on income more than actual wealth and the composition of asset portfolios. Among those who investigate the role asset composition plays on determining well-being, a more macro (rather than micro) approach is usually taken, comparing static levels of wealth and their variations as their main explanatory factors influencing health. 

Given the number of confounding mediators and moderator factors that are present, it is extremely difficult to classify all the relevant literature into separate pieces that translate income to wealth and health, and health to well-being, either on levels or change of levels. At any rate, some studies have mainly focused on (i) the pure income-wealth-health link [9,10,11], (ii) the relation between net wealth (i.e., gross wealth minus debt) and its composition and health [12,13,14], and (iii) the impact of over-indebtedness (net wealth burden) and individual health status with regard to emotional states associated with depression, stress, anxiety and mental health [15,16], declining physical health [17], unhealthy behavior [18,19,20] and suicidal tendencies [21,22]. 

In this body of literature, living conditions may be a first mediator. Aittomäki et al. explored how the wealth of an individual or a household affects health through the effects on living conditions as well as through social comparison and experiences of deprivation [9]. From a survey of Finnish men and women aged from 45 to 67 years, all of whom were civil servants, and in a period before the crisis (2001–2007), they found household wealth to have a strong and consistent association with self-rated health, with poor health decreasing as wealth increased. The relationship was only partly attributable to the association of wealth with employment status, household income, work conditions, and health-related behavior. The association of household income with self-rated health was greatly diminished when taking into account employment status and wealth, and even further attenuated by work conditions. The insufficiency of current income as the only measure of material welfare and the conditions associated with long-term accumulation of material welfare may be a significant aspect of the causal processes that lead to socioeconomic inequalities in ill health. Benzeval and Judge pay particular attention to the role of long-term income as a proxy for wealth, to conclude that wealth is more important for health than current income, and persistency is more harmful to health than occasional episodes [23]. 

Psychological elements may be moderators of the former factors. Bridges and Disney show that although there is a positive association between subjective measures of financial well-being and psychological well-being, individuals differ in their psychological response to objective household financial situations [16]. Dietz and Haurin focus their attention on the effects of real assets to note that homeowners are happier and healthier than non-owners. However, the correlation between both variables has some clear confounding factors, such as income and education [24]. At any rate, homeowners report higher self-ratings on their physical health even after controlling for age and socioeconomic factors.

With regard to net wealth variations, Gathergood analyzes over-indebtedness to conclude that individuals exhibiting problems repaying their debt obligations also exhibit much poorer psychological health [25]. Using individual-level UK panel data, local house price movements exogenous to individual households are used to establish the causality from problem mortgage debt to psychological health. Interestingly, there seems to exist a sort of ‘social norm effects’ of debt (how extended, how general these problems are) by investigating local bankruptcy and repossession rates. 

On the importance of asset composition, Berger et al., analyze data from 1987 to 1994 from the USA National Survey of Families and Households in a series of regression models, some of which included individual-specific fixed effects, to estimate associations of particular types and levels of debt with adult depressive symptoms [14]. Results suggest that household debt is positively associated with greater depressive symptoms. However, this association appears to be driven by short-term (unsecured) debt; they found little evidence of associations with depressive symptoms for mid- or long-term debt. In similar terms, Brown et al., explore the association between debt and psychological well-being amongst heads of households using the British Household Panel Survey [13]. Household heads who have outstanding (non-mortgage) credit, and who have higher amounts of such debt, are significantly less likely to report complete psychological well-being. No such significant association is found in the case of mortgage debt. Their results highlight the psychological cost associated with the consumer credit culture in Britain.

Turunen and Hiilamo survey a sample of 33 peer-reviewed studies [26]. From the results, they show serious health effects related to indebtedness. Individuals with unmet loan payments had suicidal ideation and suffered from depression more often than those without such financial problems. Unpaid financial obligations were also related to poorer subjective health and health-related behavior. In a similar vein, Richardson et al., conclude that those with depression are more than twice as likely to be in debt; 42% of those in debt have a mental disorder compared to 18% with no debt. Furthermore, 25% of those with a mental disorder are in debt, compared to 9% in those who are healthy [27].

On the effects of wealth changes, Pool et al., explores how a sudden loss of wealth—a negative wealth shock—may take a significant mental health toll and leave fewer monetary resources for health-related expenses [4]. With limited years remaining to regain lost wealth in older age, the health consequences of these negative wealth shocks may be long-lasting. Among US adults aged 51 years and older, a loss of wealth over two years was associated with an increased risk of all-cause mortality. By estimating how the marginal utility of consumption varies with health, from data on permanent income, health in older people and people of a similar elderly age, and proxy for utility with measures of subjective well-being, Finkelstein et al., find that the marginal utility of consumption declines as health is felt to deteriorate [5]. This has a substantial effect on the optimal levels of health insurance benefits and life-cycle savings. This latter issue is taken up by Liu and Menegatti [6]. They study how health and wealth investments react to the presence of random returns, distinguishing the case where only the level of health investment is chosen from the case where both health and wealth investments are chosen. The authors show that this reaction depends mainly on certain features of preferences: cross-prudence/imprudence in wealth, cross-prudence/imprudence in health, and the value of the indices of relative prudence in wealth and in health being larger or smaller than the threshold in determining optimal choices.

Finally, there is a wide range of papers that focus on risk aversion and household characteristics as these two factors shape the impact on health in terms of reflecting individual attitudes. See Riley and Chow for how risk-taking changes with wealth [28], Pälson with age [29], Bellante and Saba across the life cycle [30], Bellante and Green on the type of portfolio [31], Albert and Duffy by observing intergenerational attitudes [32], and Bommier and Rochet on as individuals increase their share of wealth held in risky assets when they age [33]. 

From an aggregate national perspective, Bover et al., present the differences across the Euro area countries of well-being and the distributions of various measures of debt conditional on household characteristics, including the probability of holding debt, the amount of debt held and, in the case of secured debt, the interest rate paid on the main mortgage [34]. In fact, the patterns of secured and unsecured debt outcomes vary markedly across countries. Clayton et al., investigate the relationship between aggregate household debt and aggregate health outcomes across 17 European countries over the period 1995 to 2012 [12]. They estimate an instrumental variable (GMM) model to address possible reverse causality concerns. Aggregate household debt affects health outcomes, and this varies by the maturity of debt. Long-term (rather than short/medium term) unsecured debt and mortgage debt are associated with poorer health outcomes. 

Finally, Schwandt analyzes how wealth shocks affect the health of older people in developed countries [3]. By exploiting the booms and busts in the US stock market as a natural experiment that generated considerable gains and losses in the wealth of stock-holding retirees, the author links wealth shocks as the interaction of stock holdings with stock market changes, to find that wealth shocks predict wealth changes and they strongly affect health outcomes. A 10% wealth loss leads to an impairment of 2–3% of a standard deviation in physical health, mental health, and survival rates.

For Spain, the single study we have found is that of Blázquez and Budría [7]. They construct some measures of debt strain such as debt-to-income ratios. Their paper differentiates between mortgage and non-mortgage debts and spans to 2011, thus including the housing bubble and the beginning of the financial crisis. The results, based on a random effects model, show that non-mortgage debt payments and debt arrears are negatively related to people’s health. However, Blázquez and Budría approach debt rather than wealth and the period does not cover the end of the crisis. In addition, they ignore the structural change associated with the crisis, as results from a previous paper of ours [8]. Moreover, they do not account for the fact that the distribution of wealth is heavily skewed, which is why the data source from where they obtain the data (the Spanish Survey of Household Finances) oversamples the wealthiest households [8]. 

In summary, after examining the literature, our hypothesis here is that wealth has an observed, rather robust protective role for well-being and via this for health when income falls. Likewise, the increase in the burden of those indebted (net wealth changes) may move in the opposite direction, thus creating anxiety, stress, increased cardiovascular risk and health deterioration. In addition, we postulate that the impact the rate of change and volatility have on health may be very relevant, even more so than rates and levels, and that the portfolio composition of debt is also important: financial versus real estate assets, type and liquidity of the financial assets, credit card use, and debt liability and maturity. In addition, portfolio compositions differ for age cohorts, household composition, and perhaps previous health status (to control for reverse causality) as they vary among countries [8,22].

Spain is a good case study to test our hypotheses for at least two reasons. First, the share of individuals owning a house is the largest in the European Union, which in itself has been considered a stabilizing factor of wealth distribution. House ownership (along with pension entitlement) protect adults and older people, and its evolution is rather stable due to the role of inheritance on a dynastic basis. However, the variation in housing prices and mortgage interest rates may have created anxiety in comparison with the volatility of other assets. This may have to do with variations and levels of over-indebtedness and the burden of other existing debts with regard to current income and changes in the saving capability of individuals. First, our data (the Spanish Survey of Household Finances) shows how wealth stabilizes consumption for the young cohorts through indebtedness. The change in average household consumption due to income changes is lower in households with more wealth in those families where the head of household is under 55 years old. Specifically, in a household with little relative wealth (the one with only 5% of the households below) and in which the head of the family is 30 years old, a 1% decrease in income generates a 0.5% drop in consumption. The decrease is lower (0.1%) in households with the highest wealth (those with less than 95% of households). However, in households over 55 years of age, the drop in consumption before a 1% decrease in income is always around 0.3%, regardless of wealth level [35]. Second, the degree of resilience for the link between wealth fluctuations and health may be approached with data from the past financial crisis. In Spain, this has implied a loss of per capita income of around a tenth of its previous level with a reduction in the value of some dwellings being even higher; estimated as a 37% average loss [18,19]. A sensible hypothesis is to test whether the economic and financial crisis has affected health as a result of the changes experienced in net wealth. 

Our objective in this paper is to evaluate the association between the variations in income and wealth (both in aggregate and in split between real estate and financial wealth) and self-perceived health in Spain. We take advantage of a rich new data set covering the financial crisis Spain suffered, which, in turn, created a plethora of inferences on its effects on Spanish health. The financial crisis was directly triggered by the collapse of the housing bubble in the United States in 2006, which caused a mortgage loan crisis in the last quarter of 2007. The crisis took place between 2007 and 2013, albeit with some differences between countries in its timing and scale. In Spain, the financial crisis began in the first quarter of 2009 and ended in 2014. 

We meet our objective by considering levels of wealth and the variation of its two main components – real estate wealth and financial wealth, gross and net wealth. Within each of these categories, between gross and net wealth—on self-perceived health. As the explanatory variables of control, we include variables at the family level: (i) savings rate; (ii) number of family members; (iii) number of family members who work; (iv) property regime of the family dwelling (not owned by the family-reference category—or owned by the family) and (v) proportion of real estate wealth over total wealth. We also include control variables at the individual level: sex, age, educational level, occupation, and marital status.

The remainder of this paper is structured as follows. Section 2 and Section 3, respectively, describe the data and methodology used for our analysis. Results are presented in Section 4, and Section 5 concludes the paper.

## 2. Data

### 2.1. Data Sources 

We use data from the Spanish Survey of Household Finances (SSHF henceforth), a longitudinal database [36] collecting socioeconomic information on a random sample of the Spanish population every three years and stratified by gender and age. We make use of two waves prior to the financial crisis (2005 and 2008), one during the crisis (2011) and the other at the end of it (2014). The SSHF provides detailed information on the assets, debts, income, and spending of Spanish household units. It also contains socioeconomic and demographic information and self-reported health status. The longitudinal nature allows us to follow a set of households at various points in time. More importantly, this survey is the only source of data that provides information on the wealth of Spanish families over time, allowing us to not only focus on wealth levels, but also on its composition (housing or financial assets). Our study sample includes the members of families who were interviewed in at least two waves of the SSHF and who were interviewed both before and after the crisis. We used Survey of Household Finances (EFF in Spanish) from the Bank of Spain. Public and freely accessible at: https://www.bde.es/bde/en/areas/estadis/Otras_estadistic/Encuesta_Financi/. This article does not contain any studies performed by any of the authors using human participants or animals.

### 2.2. Data Description

#### 2.2.1. Outcome Variable

Respondents are asked to rate their health in one of the following five categories: ‘very good’, ‘good’, ‘fair’, ‘poor’ and ‘very poor’. We dichotomize their responses into two categories: fair, poor and very poor (taking value 0, which we will refer to as ‘poor health’) and very good and good (value 1, and labelled ‘good health’ in the rest of our analysis).

#### 2.2.2. Explanatory Variables

Our key explanatory variables are the variation in gross wealth. In addition to this, we consider the variation of its two main components—real estate wealth and financial wealth; and within each of these categories, between gross and net wealth. We also consider the variation in total debt, in order to approach the net wealth of the household. We measure variation in real income in order to compare it with the variation in the wealth variables.

As the explanatory variables of control, we include variables at the family level:

(i) savings rate
(ii) number of family members
(iii) number of family members who work
(iv) property regime of the family dwelling (not owned by the family-reference category-or owned by the family)
(v) proportion of real estate wealth over total wealth

We also include control variables at the individual level: sex, age, educational level, occupation, and marital status.

Finally, we include the year of the survey wave (2005, 2008, 2011, and 2014). Using a smoothing spline, we allow the relationship between the explanatory variable of interest and the response variable to be non-linear. In particular, we include a random effect associated with the year of the wave, using a random walk of order 1 as a smoother. 

The relationships between the explanatory variables (both those of interest and the controls) with probability of declaring good or very good health are not linear at all. We approximated the non-linearity categorizing the variables (see Table 1). We prefer not to use nonparametric or other parametric approximations (for example polynomials), because their use makes interpretation very difficult. 

## 3. Methods 

We specify two generalized linear mixed models with a binomial response and a logistic link (equivalent to mixed logistic regressions), in one, we include gross wealth, and in the other, we decompose gross wealth into its components (real estate and financial wealth).

(1a) $$log\left(\frac{Prob\left(Y_{ijt}=1\right)}{1-Prob\left(Y_{ijt}=1\right)}\right)=\alpha_{0i}+\alpha_{0j}+\sum_{k=2}^{5}\beta_{1k}Gross\;{wealth}_{ijt,k}+\sum_{k=2}^{5}\gamma_{1k}Real\;{income}_{ijt,k}+\;\sum_{k=2}^{5}\gamma_{2k}Total\;{debt}_{ijt,k}+\sum_{k=2}^{3}\gamma_{3k}Proportion\;real\;{state}_{ijt,k}+\sum_{k=2}^{4}\gamma_{4k}Savings\;{rate}_{ijt,k}+\gamma_{5}{sex}_{ij}+\;\sum_{k=2}^{5}\gamma_{6k}{age}_{ijt,k}+\sum_{k=2}^{4}\gamma_{7k}Educational\;{level}_{ijt,k}+\sum_{k=2}^{8}\gamma_{8k}{Occupation}_{ijt,k}+\sum_{k=2}^{4}\gamma_{9k}Marital\;{status}_{ijt,k}+\;\gamma_{10}Property\;{ownership}_{ijt}+\tau_{t}$$

(1b) $$log\left(\frac{Prob\left(Y_{ijt}=1\right)}{1-Prob\left(Y_{ijt}=1\right)}\right)=\alpha_{0i}+\alpha_{0j}+\sum_{k=2}^{5}\beta_{1k}Real\;state\;{wealth}_{ijt,k}+\;\sum_{k=2}^{5}\beta_{2k}Financial\;{wealth}_{ijt,k}+\sum_{k=2}^{5}\gamma_{1k}Real\;{incom}_{ijt,k}+\sum_{k=2}^{5}\gamma_{2k}Total\;{debt}_{ijt,k}+\;\sum_{k=2}^{3}\gamma_{3k}Proportion\;real\;{state}_{ijt,k}+\sum_{k=2}^{4}\gamma_{4k}Savings\;{rate}_{ijt,k}+\gamma_{5}sex_{ij}+\;\sum_{k=2}^{5}\gamma_{6k}{age}_{ijt,k}+\sum_{k=2}^{4}\gamma_{7k}Educational\;{level}_{ijt,k}+\sum_{k=2}^{8}\gamma_{8k}{Occupation}_{ijt,k}+\;\sum_{k=2}^{4}\gamma_{9k}Marital\;{status}_{ijt,k}+\gamma_{10}Property\;{ownership}_{ijt}+\tau_{t}$$

where the subindexes i and j denotes the study subject and the family to which the subject belongs, respectively; *t* denotes the wave of the survey; k the category of the variable (see Table 1); Y denotes the response variable (1 for good and very good health, 0 otherwise); $\alpha_{0i}$, $\alpha_{0j}$ and $\tau_{t}$ denotes random effects (explained below); and βs, γs are the coefficients of the explanatory and control variables, respectively ($e^{\beta }\;\mathrm{and}\;e^{\gamma }$ are the odds ratios, OR, associated with each of them).

To interpret the ORs this formula can be used, (OR − 1) × 100. Thus, if the OR is equal to 1.12, for example, a 12% more probability will be interpreted ((1.12 − 1) × 100 = 12%), while if the OR is equal to 0.88, it will be interpreted as 12% less likely ((0.88 − 1) × 100 = −12%).

We included three random effects in the models. First, $\alpha_{0i}$ and $\alpha_{0j}$, random effects indexed on the individual and on the family to which the individual belonged. These random effects were unstructured (independent and identically distributed random effects), and captured individual and familial heterogeneity, that is to say, unobserved confounders specific to the small area and invariant in time. Heterogeneity (individual and family) contains those unobserved confounders (associated with the individual and the family) that could also influence the probability of declaring good health.

Second, in the model we included $\tau_{t}$, a structured random effect (random walk of order one, rw1). Following the integrated nested Laplace approximations (INLA) approach [37,38] when, as in our case, the random effects are indexed on a quantitative variable (the year of the survey wave), they can be used as smoothers to model non-linear dependency on covariates in the linear predictor. That is, we assume that the probability of declaring good health varies in a non-linear way between the different waves of the survey.

Following the INLA approach, random effects were defined using a multivariate Gaussian distribution with a zero mean and precision matrix kΣ, where *k* was a constant and Σ was a matrix that defined the dependence structure of the random effects [37,38]. In unstructured random effects (iid) Σ was a diagonal matrix of 1s; and in random walk random effects Σ was defined assuming that increments (in rw1, $\Delta u_{i}=u_{t}-u_{t-1}$) followed a Gaussian distribution with zero mean and a constant precision *k* [39].

The distribution of wealth is heavily skewed. For this reason, the SSHF oversamples the wealthiest households. This is done to ensure that its sample is representative not only of the Spanish population as a whole, but also of the aggregate wealth of the Spanish economy. We corrected that oversampling by including in the models the weights provided by the SSHF itself in each of its waves.

We estimate the models {1a} and {1b}, both without stratifying and stratifying by the age group of the reference person of the family (under 35 years, 35 to 44 years, 45 to 54 years, 55 to 64 years, 65 to 74 years, 75 or more years).

Given the complexity of our model, we perform inferences using a Bayesian framework. In particular, we followed the Integrated Nested Laplace Approximation (INLA) approach [37,38], within a (pure) Bayesian framework. We used priors that penalize complexity (called PC priors). These priors are robust in the sense that they do not have an impact on the results and, in addition, they have an epidemiological interpretation [40]. 

All analyses were made with the free software R (version 3.6.0) [41], through the INLA package [37,38,42].

## 4. Results

In the sample, we included a total of 36,421 individuals belonging to 13,646 families, observed during at least two waves (out of four possible). This random sample represented a population of 20,038,899 individuals and 7,109,404 families.

In Table 2, Table 3 and Table 4 we show the descriptive statistics of the response variables. A clear age gradient in wealth can be observed as well as a rather biased distribution (differences between mean and median values) with unequal distributions increasing with age (younger being more equally poor). As expected, real estate is much more concentrated among older populations, while financial wealth goes to the over 55 to 75-year-old population (i.e., the baby boom generation), with the under 35 s in the poorest situation. Debt is particularly high for the 35 years or younger age group, whereas this decreases in the 75-year-old group. Conversely, real income moves in the opposite direction i.e., those with lower incomes are those who are most in debt. Unequal distributions, as observed by the differences between the medians and the means, increase with age and wealth (not so much on real income probably as a consequence of the impact of pensions for retirees), showing actual levels equal to or even above those from the 35 years and younger generation. Static associations prove a clear gradient between good and very good health, and fair, poor, and very poor health with total debt (up and down); albeit not so much on the rest of the variables. Financial wealth distribution is highly skewed in self-rated health, as it is for total debts.

In Table 3, Table 4, Table 5, Table 6, Table 7 and Table 8, in addition to the OR and their credibility intervals at 95% (95% ICr, from now on), the probability of the parameter estimator (the log (OR)) as an absolute value being more than 0 is also shown. Unlike the *p*-value in a usual environment (i.e., frequentist), this probability allows us to make inferences about possible associations.

In terms of wealth variations, and as can be seen in Table 5, the odds ratios (OR) of the wealth variables are quite similar (with the exception of the odds ratios of the proportion of real estate wealth over total wealth). However, the statistical significance differs. Thus, in gross wealth, it is the decrease that is statistically associated with the probability of declaring good or very good health (with a reduction in that probability between 4.37% and 12.91%—these figures were calculated from the formula (OR − 1) × 100, thus (0.95647 − 1) × 100 = 4.37% and (0.87095 − 1) × 100 = 12.91%, with a lower probability, the greater the reduction in gross wealth. For the gradients above 40% and between 10 and 40%, the intervals overlap perhaps due to the lower numbers for those who may have seen their gross wealth increase by more than 40% during the crisis. As mentioned earlier, in the exercises we control for gender, debt levels, age, level of education, occupation, marital status, number of family members, number of family members who work, savings rate, property ownership regimen, proportion of real estate over total wealth, and year of the survey. However, both for real estate and financial wealth it is their growth that is associated with a greater probability of declaring good or very good health. The difference is that, in real estate, wealth the effect on probability is greater (an increase of 24.34%) and is especially significant when there is an increase of 50% or greater (the increase in probability is 8.5% and significant only at 90%). Real wealth increases appear to be more robust for improving the assessment of self-perceived health. In financial wealth, the increase in probability (between 14.37% and 17.73%, occurs with increases from 10%. Small fluctuations seem to have an impact despite having perhaps a less reliable permanency. It should be noted, however, that, in this case, the two credibility intervals overlap and, therefore, it cannot be said that the two ORs are different from 95%. 

Total debt fluctuations exhibit the expected gradient. Decreases in total debt from 10% increase the probability of declaring good or very good health between 47.58% and 67.64% while increases from 10% decrease it between 36.52% and 42.32% (in both cases the credibility intervals overlap) (Table 6). Lack of symmetry shows the importance of debt alleviation more than debt contraction. A reduction in real income equal to or greater than 40% decreases the probability of declaring good or very good health by 18.61%, whereas an increase equal to or greater than 50% increases it by 62.75%. Increased cash availability has a stronger effect for better self-assessed health than cash reductions do for worse self-assessed health.

The results of the stratification by age groups of the family reference person are found in Table 4, Table 5, Table 6, Table 7 and Table 8.

In general, the observed associations in all the age groups and fluctuation intervals were to be expected. For changes in gross wealth in general, the positive or negative impacts are more pronounced with increasing age. There is a higher probability of declaring good/very good health with ageing for larger positive variations. Meanwhile, in the ‘likely to be pensioners’ group, a growth in wealth greatly increases this probability for the younger individuals more than the rest. Decreases in gross wealth are those that are associated with decreases in the probability of health in families whose reference person is over 44 years old (Table 7 and Table 8). For families with a younger reference person, however, only increases in gross wealth are associated with increases in probability. Note that these increases are much greater than the decreases in families with a reference person over 44 years of age.

This is accentuated for real estate—increases 10%–50%—and more are strongly associated with increases in the probability of rating health as good or very good (Table 9 and Table 10). Unlike gross wealth, in families whose reference person is 75 years or older, variations in real estate wealth are not associated with the probability of declaring good or very good health (Table 9). Also note that, in this case, in addition to the general behavior, in families whose reference person is between 55 and 64 years old and those who are 35 years old or younger, decreases in real estate wealth greater than or equal to 50% decrease the probability of declaring good and very good health.

Regarding the ORs of the variations in financial wealth, although the behavior is similar, (i.e., that without stratification and that in other wealth variables), the statistical meanings are highly variable between the age groups of the reference person in the family (Table 11 and Table 12). It should be noted that in families with a reference person aged 75 or over and 35 or under, both increases and decreases in financial wealth are statistically significantly associated with increases and decreases, respectively, in the probability of declaring good or very good health. The gradient of the associations again is the expected one, with lower ranks of values as age increases and more extreme values for fluctuations above 50%.

The behavior of the changes in total debt stratified by the age groups of the reference person (Table 13 and Table 14), follows a similar pattern to that for real and financial assets but with the opposite sign. Probability of declaring good or very good health shows a higher ‘elasticity’ for younger individuals and higher ranks and more resilience for the older age groups. This closely resembles that of the non-stratified changes (Table 3) in families in which the reference person is between 55 and 74 years old and between 35 and 44 years old (especially between 55 and 64 years old). Note that, as in the case of real estate wealth, in the families whose reference person is 75 years or older, variations in total debt are not associated with the probability of declaring good or very good health (Table 13).

Finally, regarding variations in real income, age cohorts are less sensitive to changes although the gradient is the one expected. This may explain why the associations with the variation in income are extreme (the two tails), and the effects are larger and the difference between them wider than in the variations in the wealth variables. For the very young, they have a good or poor health quite independent of the flow fluctuations of income in a less elastic way than for real assets. As in the unstratified case, increases and decreases at the ends of the tails are associated with the probability of declaring good or very good health in families whose reference person is between 55 and 74 years old. Conversely, these probabilities are associated only with increases in families with a reference person of 75 years or older and only with decreases in families whose person of reference persons is under 55 years of age (Table 15 and Table 16).

## 5. Discussion

The waves of data used in our analysis cover a period of time in which there were major economic fluctuations between 2005/2008/2011/2014. 

In 2005, when the survey was carried out for the first time, the economy was doing well but in this very same year, the deceleration of the real GDP growth started after a decade of important non-stop increases. In 2008 Spain suffered from negative growth, but the worst was yet to come. As people started to become deeply concerned, the savings rate increased by a precautionary 4 points, although consumption held thanks to unemployment subsidies. In 2011, income and consumption dropped by 6 percentage points of GDP compared to 2005, but even so, it was not yet close to its lowest level—minus 9 points—in 2013. This occurred when unemployment subsidies vanished and although other non-contributive measures were introduced, they were at very much lower levels in comparison. The period from 2011 to 2014 was very unsettled and commenced with a very depressing double dip (meanwhile, the EU was recovering). One and half years later, there was an improvement thanks to, initially, public consumption (and larger deficits) and later on private consumption: the resulting 3-point fall in the savings rate over this period indicated a somewhat more optimistic perspective overall. Finally, in 2014 the future began to look more positive as employment started to be created once again. 

The worst losses in disposable income occurred between 2007 and 2013, which saw the greatest increase in unemployment and a reduction in disposable income for all types of families: m18% on average with regard to 2007. After all this, those relatively worse off (above negative average) are single individuals less than 30 years old (loss of 35%), those between 30 and 65, and couples with two children (−20%). We find monoparental families with at least one child are also in a very poor situation (−19%). Couples with a single child and couples with three or more children (likely to already be wealthy on average terms) are below the average, albeit better situated than the other groups, but below the levels of the departure year. In a more positive situation are those over 65 (with a positive plus 1%). 

We extend the analysis of Blazquez and Budria [7] to a period, which fully captures the financial crisis under some dramatic changes. We improve the estimation method with regard to data bias (over-representation of the wealthy groups), and we focus on wealth rather than over indebtedness (the debt to income ratios during the economic expansion of the housing bubble). We analyze real estate versus financial asset composition (rather than mortgage/non-mortgage debt). Blazquez and Budria conclude that non-mortgage debt payments and debt arrears are negatively related to people’s health. With regard to the existing literature, we offer a more complete picture of wealth variation effects on self-perceived health, and we analyze the three major factors at play. The influence of (i) not just income levels but net wealth changes reflect in health and optimism—these move with age with aged individuals being more sensitive to these changes, and income increases have a larger incidence on self-assessed health than equal amounts in income reductions; (ii) the composition of the net wealth of individuals (financial versus real assets)—this has also had a bearing as individuals have been differently affected by the shocks in the economic crisis and risk levels have diverged on both types of properties, as do random losses associated with them, with real more than financial wealth showing an association to self-perceived health; and (iii) age—since older people have the ‘safety net’ of their pensions and so are quite impermeable to the crisis i.e., real wealth changes have a smaller impact on the self-perceived health of aged individuals. Meanwhile, the individual reactions in terms of consumption and savings, given any level of income and wealth, and which would complete the picture, have not been considered here due to lack of data—despite the fact they possibly play a role as well. 

In general, we observe that the associations with the variation in income are extreme, and their differences are wider than in the variations of the wealth variables. Young individuals have good or poor self-perceived health quite independent of the flow fluctuations of income, and in a less elastic way in particular for real assets, than older individuals do.

An interesting point for future research would be to compare our results with some other countries’ financial household surveys, which, like the Spanish one, follow the methodology of the European Central Bank, and extend the evaluation with the new waves of survey data soon to be made available. 

## Figures and Tables

**Table 1:** Categorization of the variables.

Variable	Categorization	Notes	*N*
Gross wealth		The categories correspond to the quintiles	
	Decrease greater than or equal to 40%	1st quintile	5298
	Decrease between 10% and 40%	2rd quintile	5293
	Variation between −10% and 10% [Reference category]	3rd quintile	5294
	Growth between 10% and 50%	4th quintile	5298
	Growth greater than or equal to 50%	5th quintile	5293
Real estate wealth		The categories correspond to the quintiles	
	Decrease greater than or equal to 40%	1st quintile	4860
	Decrease between 10% and 40%	2nd quintile	4875
	Variation between −10% and 10% [Reference category]	3rd quintile	4848
	Growth between 10% and 50%	4th quintile	4860
	Growth greater than or equal to 50%	5th quintile	4861
Financial wealth		The categories correspond to the quintiles	
	Decrease greater than or equal to 40%	1st quintile	5092
	Decrease between 10% and 40%	2nd quintile	5029
	Variation between −10% and 10% [Reference category]	3rd quintile	5063
	Growth between 10% and 50%	4th quintile	5062
	Growth greater than or equal to 50%	5th quintile	5061
Real income		The categories correspond to the quintiles	
	Decrease greater than or equal to 40%	1st quintile	5330
	Decrease between 10% and 40%	2nd quintile	5331
	Variation between −10% and 10% [Reference category]	3rd quintile	5331
	Growth between 10% and 50%	4th quintile	5329
	Growth greater than or equal to 50%	5th quintile	5332
Total debt		The categories correspond to the quintiles	
	Decrease greater than or equal to 30%	1st quintile	2001
	Decrease between 10% and 30%	2nd quintile	2003
	Variation between −10% and 10% [Reference category]	3rd quintile	2000
	Growth between 10% and 50%	4th quintile	2003
	Growth greater than or equal to 50%	5th quintile	2000
Proportion of real estate wealth on total wealth		The categories correspond to the terciles	
	Decrease greater than or equal to 10%	1st tercile	8361
	Variation between −10% and 10% [Reference category]	2nd tercile	8152
	Growth greater than or equal to 10%	3rd tercile	7791
Savings rate	1st quartile [Reference category]		9079
	2nd quartile		9080
	3rd quartile		9078
	4th quartile		9080
Sex	Male [Reference category]		16,071
	Female		20,350
Age	Under 35 years [Reference category]		11,791
	35–44 years		3908
	45–54 years		4935
	55–64 years		5010
	65–74 years		5237
	75 or more years		5540
Educational level	Insufficient instruction [Reference category]	Without studies or incomplete primary Including vocational training	557
	Primary		12,805
	Secondary		9458
	University		7552
	Working as an employee [Reference category]		7426
Occupation	Self-employed		2935
	Unemployed		2446
	Retired		6210
	Disabled		891
	Student		3868
	Homemaker		7388
	Other situations		940
	Single [Reference category]		8997
Marital status	Married or with a partner		18369
	Divorced or separated		1510
	Widowed		3228
	No [Reference category]		4945
Property ownership	Yes		31,476
		

The total *n* for each variable does not coincide in all cases, due to the presence of missing observations.

**Table 2:** Descriptive statistics.

All Subjects	Mean (Std Deviation)	Median (1st Quartile-3rd Quartile)
Gross wealth		
All subjects	€396 (€89)	€252 (€100–€252)
75 years or older	€513 (€1415)	€303 (€165–€606)
65–74 years	€502 (€1392)	€266 (€154–€523)
55–64 years	€594 (€1535)	€352 (€191–€578)
45–54 years	€429 (€825)	€284 (€172–€473)
35–44 years	€310 (€424)	€229 (€154–€350)
35 years or younger	€258 (€339)	€201 (€132–€307)
Real estate wealth		
All subjects	€305 (€425)	€220 (€141–€353)
75 years or older	€428 (€572)	€300 (€143–€466)
65–74 years	€376 (€575)	€251 (€147–€430)
55–64 years	€419 (€575)	€376 (€165–€478)
45–54 years	€331 (€453)	€242 (€150–€389)
35–44 years	€248 (€214)	€204 (€141–€297)
35 years or younger	€218 (€215)	€178 (€119–€264)
Financial wealth		
All subjects	€45 (€345)	€9 (€2–€31)
75 years or older	€55 (€2,877)	€9 (€1–€38)
65–74 years	€90 (€957)	€7 (€2–€32)
55–64 years	€83 (€465)	€14 (€3–€59)
45–54 years	€42 (€284)	€12 (€3–€39)
35–44 years	€31 (€121)	€8 (€2–€24)
35 years or younger	€20 (€57)	€4 (€1–€14)

In thousands of euros. Average of the 4 waves.

**Table 3:** Descriptive statistics.

All subjects	Mean (Std Deviation)	Median (1st Quartile-3rd Quartile)
Total debt		
All subjects	€77 (€126)	€50 (€18–€106)
75 years or older	€57 (€163)	€13 (€3–€80)
65–74 years	€48 (€106)	€16 (€6–€60)
55–64 years	€56 (€223)	€26 (€9–€72)
45–54 years	€74 (€91)	€45 (€17–€101)
35–44 years	€86 (€92)	€62 (€25–€120)
35 years or younger	€102 (€97)	€84 (€42–€135)
Real income		
All subjects	€48 (€52)	€36 (€24–€56)
75 years or older	€37 (€90)	€31 (€17–€40)
65–74 years	€45 (€50)	€35 (€23–€54)
55–64 years	€59 (€65)	€45 (€28–€71)
45–54 years	€53 (€57)	€40 (€27–€59)
35–44 years	€43 (€37)	€35 (€24–€52)
35 years or younger	€38 (€46)	€30 (€20–€45)

In thousands of euros. Average of the 4 waves.

**Table 4:** Descriptive statistics.

All Subjects	Mean (Std Deviation)	Median (1st Quartile-3rd Quartile)
Gross wealth		
Good/very good self-perceived health	€397 (€896)	€253 (€159–€433)
Fair/poor/very poor self-perceived health	€391 (€879)	€251 (€143–€412)
Real estate wealth		
Good/very good self-perceived health	€307 (€346)	€220 (€141–€362)
Fair/poor/very poor self-perceived health	€305 (€434)	€220 (€132–€349)
Financial wealth		
Good/very good self-perceived health	€45 (€356)	€9 (€2–€31)
Fair/poor/very poor self-perceived health	€44 (€230)	€8 (€2–€31)
Total debt		
Good/very good self-perceived health	€79 (€129)	€53 (€20–€109)
Fair/poor/very poor self-perceived health	€59 (€92)	€28 (€7–€81)
Real income		
Good/very good self-perceived health	€48 (€52)	€37 (€25–€56)
Fair/poor/very poor self-perceived health	€43 (€50)	€32 (€21–€49)

In thousands of euros. Average of the 4 waves.

**Table 5:** Association between the variation in wealth and real income and good and very good self-perceived health.

All Subjects	Odds Ratio	95% Credibility Interval		Prob (|log(OR)|) > 0
Gross wealth				
Gross wealth [Variation between −10% and 10%]				
Decrease ≥ 40%	0.95630	0.85112	1.07437	0.93519
Decrease between 10% and 40%	0.87095	0.77316	0.98101	0.98865
Growth between 10% and 50%	0.95647	0.84801	1.07868	0.76709
Growth ≥ 50%	1.05326	0.93449	1.18700	0.80202
Real estate wealth				
Real state wealth [Variation between −10% and 10%]				
Decrease ≥ 40%	0.99386	0.87933	1.12320	0.54050
Decrease between 10% and 40%	0.95796	0.85401	1.07644	0.76760
Growth between 10% and 50%	1.08520	0.96019	1.22636	0.90478
Growth ≥ 50%	1.24341	1.10361	1.40078	0.99983
Financial wealth				
Financial wealth [Variation between –10% and 10%]				
Decrease ≥ 40%	0.91120	0.82021	1.01219	0.85914
Decrease between 10% and 40%	0.95904	0.83313	1,11753	0.70337
Growth between 10% and 50%	1.14374	1.01374	1.29056	0.98556
Growth ≥ 50%	1.17728	1.04600	1.32517	0.99664

Adjusted by sex, age, level of education, occupation, marital status, number of family members, number of family members who work, saving rate, property ownership, proportion of real estate over total wealth, and year of the survey. Reference category between brackets

**Table 6:** Association between the variation in wealth and real income and good and very good self-perceived health.

All Subjects	Odds Ratio	95% Credibility Interval		Prob (|log(OR)|) > 0
Total debt				
Total debt [Variation between −10% and 10%]				
Decrease ≥ 30%	1.67644	1.29506	2.17058	0.99999
Decrease between 10% and 30%	1.47576	1.26240	1.72542	0.99999
Growth between 10% and 50%	0.63480	0.52533	0.76696	0.99999
Growth ≥ 50%	0.57683	0.47212	0.70466	1.00000
Real income				
Real income [Variation between −10% and 10%]				
Decrease ≥ 40%	0.81389	0.72268	0.91652	0.99968
Decrease between 10% and 40%	0.93348	0.82698	1.05359	0.86844
Growth between 10% and 50%	1.02920	0.91283	1.16028	0.68015
Growth ≥ 50%	1.62754	1.44338	1.83501	1.00000

Adjusted by sex, age, level of education, occupation, marital status, number of family members, number of family members who work, saving rate, property ownership, proportion of real estate over total wealth, and year of the survey. Reference category between brackets

**Table 7:** Association between the variation in gross wealth and good and very good self-perceived health.

	Odds Ratio	95% Credibility Interval		Prob (|log(OR)|) > 0
Reference person 75 years or older				
Gross wealth [Variation between −10% and 10%]				
Decrease ≥ 40%	0.69478	0.52475	0.91968	0.99464
Decrease between 10% and 40%	0.79394	0.61354	0.92714	0.95088
Growth between 10% and 50%	1.12624	0.85277	1.48760	0.80001
Growth ≥ 50%	1.39162	1.07091	1.85415	0.99296
Reference person 65–74 years				
Gross wealth [Variation between −10% and 10%]				
Decrease ≥ 40%	0.72539	0.57304	0.91806	0.99630
Decrease between 10% and 40%	0.87223	0.68752	1.10633	0.87097
Growth between 10% and 50%	1.26027	0.99166	1.60197	0.94107
Growth ≥ 50%	1.13227	0.89116	1.43833	0.84511
Reference person 55−64 years				
Gross wealth [Variation between −10% and 10%]				
Decrease ≥ 40%	0.87878	0.65390	1.18071	0.90547
Decrease between 10% and 40%	0.96860	0.73199	1.28140	0.58962
Growth between 10% and 50%	1.03818	0.78107	1.37960	0.60087
Growth ≥ 50%	1.12625	0.83776	1.51371	0.78406

Adjusted by total debt, real income, sex, age, level of education, occupation, marital status, number of family members, number of family members who work, saving rate, property ownership, proportion of real estate over total wealth, and year of the survey. Reference category between brackets.

**Table 8:** Association between the variation in gross wealth and good and very good self-perceived health.

	Odds Ratio	95% Credibility Interval		Prob (|log(OR)|) > 0
Reference person 45–54 years				
Gross wealth [Variation between −10% and 10%]				
Decrease ≥ 40%	0.74056	0.55393	0.98981	0.97906
Decrease between 10% and 40%	0.87183	0.64756	1.17348	0.81820
Growth between 10% and 50%	1.09382	0.81454	1.46848	0.72385
Growth ≥ 50%	1.19488	0.88371	1.61520	0.87615
Reference person 35–44 years				
Gross wealth [Variation between −10% and 10%]				
Decrease ≥ 40%	0.80584	0.64690	1.82191	0.62142
Decrease between 10% and 40%	0.92239	0.56249	1.51203	0.62690
Growth between 10% and 50%	1.70870	1.00601	2.90041	0.97634
Growth ≥ 50%	2.41787	1.34813	4.33424	0.99848
Reference person younger than 35 years old				
Gross wealth [Variation between −10% and 10%]				
Decrease ≥ 40%	0.85287	0.44079	9.49260	0.82080
Decrease between 10% and 40%	0.95934	0.54887	12.1900	0.88537
Growth between 10% and 50%	1.70494	0.00991	102.600	0.92525
Growth ≥ 50%	3.45697	1.64097	18.8000	0.97555

Adjusted by total debt, real income, sex, age, level of education, occupation, marital status, number of family members, number of family members who work, saving rate, property ownership, proportion of real estate over total wealth, and year of the survey. Reference category between brackets.

**Table 9:** Association between the variation in real estate wealth and good and very good self-perceived health.

	Odds Ratio	95% Credibility Interval		Prob (|log(OR)|) > 0
All subjects				
Reference person 75 years or older				
Real state wealth [Variation between −10% and 10%]				
Decrease ≥ 40%	0.92142	0.72794	1.16610	0.75323
Decrease between 10% and 40%	0.89820	0.69029	1.16847	0.78919
Growth between 10% and 50%	1.08746	0.67049	1.14051	0.83971
Growth ≥ 50%	1.09231	0.71841	1.18594	0.73542
Reference person 65–74 years				
Real state wealth [Variation between −10% and 10%]				
Decrease ≥ 40%	0.67598	0.50213	0.90979	0.99521
Decrease between 10% and 40%	0.81211	0.59074	1.11613	0.90090
Growth between 10% and 50%	1.07242	0.72300	1.30757	0.57463
Growth ≥ 50%	1.20802	0.87580	1.66583	0.87511
Reference person 55–64 years				
Real state wealth [Variation between −10% and 10%]				
Decrease ≥ 40%	0.72137	0.55078	0.94501	0.99115
Decrease between 10% and 40%	0.97095	0.74988	1.25692	0.58978
Growth between 10% and 50%	1.12626	0.85486	1.48348	0.80061
Growth ≥ 50%	1.43055	1.10121	1.85797	0.99638

Adjusted by total debt, real income, sex, age, level of education, occupation, marital status, number of family members, number of family members who work, saving rate, property ownership, proportion of real estate over total wealth, and year of the survey. Reference category between brackets 95%.

**Table 10:** Association between the variation in real estate wealth and good and very good self-perceived health.

	Odds Ratio	95% Credibility Interval		Prob (|log(OR)|) > 0
Reference person 45–54 years				
Real state wealth [Variation between −10% and 10%]				
Decrease ≥ 40%	0.95684	0.85652	1.56065	0.82864
Decrease between 10% and 40%	0.95482	0.70975	1.28419	0.62132
Growth between 10% and 50%	1.08485	0.79298	1.48377	0.69403
Growth ≥ 50%	1.90723	1.39139	2.61361	0.99997
Reference person 35–44 years				
Real state wealth [Variation between −10% and 10%]				
Decrease ≥ 40%	0.91636	0.69636	2.12379	0.75381
Decrease between 10% and 40%	0.93591	0.74070	2.06139	0.79084
Growth between 10% and 50%	1.93309	1.08171	3.45213	0.98700
Growth ≥ 50%	2.29580	1.30449	4.03798	0.99804
Reference person younger than 35 years old				
Real state wealth [Variation between −10% and 10%]				
Decrease ≥ 40%	0.30865	0.09799	0.97319	0.97766
Decrease between 10% and 40%	0.46395	0.10538	2.04504	0.84461
Growth between 10% and 50%	2.91446	0.68226	12.4350	0.92557
Growth ≥ 50%	5.94600	1.78065	19.8310	0.99814

Adjusted by total debt, real income, sex, age, level of education, occupation, marital status, number of family members, number of family members who work, saving rate, property ownership, proportion of real estate over total wealth, and year of the survey. Reference category between brackets.

**Table 11:** Association between the variation in financial wealth and good and very good self-perceived health.

	Odds ratio	95% credibility interval		Prob(|log(OR)|)>0
Reference person 75 years or older				
Financial wealth [Variation between −10% and 10%]				
Decrease ≥40%	0.69404	0.48398	0.99499	0.97686
Decrease between 10% and 40%	0.81480	0.64692	1.02603	0.94962
Growth between 10% and 50%	1.32960	1.01301	1.74553	0.98024
Growth ≥ 50%	1.48642	1.15833	1.90783	0.99910
Reference person 65-74 years				
Financial wealth [Variation between −10% and 10%]				
Decrease ≥40%	0.73343	0.59720	0.90058	0.99849
Decrease between 10% and 40%	1.04381	0.78156	1.39373	0.61334
Growth between 10% and 50%	1.22751	0.96767	1.55742	0.94486
Growth ≥ 50%	1.31762	1.03945	1.67057	0.98884
Reference person 55–64 years				
Financial wealth [Variation between −10% and 10%]				
Decrease ≥ 40%	0.91969	0.73098	1.15690	0.76384
Decrease between 10% and 40%	0.89049	0.68625	1.15527	0.80976
Growth between 10% and 50%	1.15422	0.84700	1.57248	0.81780
Growth ≥ 50%	1.20540	0.91663	1.58478	0.90930

Adjusted by total debt, real income, sex, age, level of education, occupation, marital status, number of family members, number of family members who work, saving rate, property ownership, proportion of real estate over total wealth, and year of the survey. Reference category between brackets.

**Table 12:** Association between the variation in financial wealth and good and very good self-perceived health.

	Odds Ratio	95% Credibility Interval		Prob (|log(OR)|) > 0
Reference person 45–54 years				
Financial wealth [Variation between −10% and 10%]				
Decrease ≥ 40%	0.82131	0.62642	1.07658	0.92364
Decrease between 10% and 40%	0.88774	0.66323	1.18798	0.78953
Growth between 10% and 50%	1.20579	0.79632	1.82517	0.81130
Growth ≥ 50%	1.46912	1.09878	1.96475	0.99537
Reference person 3–44 years				
Financial wealth [Variation between −10% and 10%]				
Decrease ≥ 40%	0.96903	0.80223	2.00646	0.84549
Decrease between 10% and 40%	0.98545	0.47513	2.04251	0.51690
Growth between 10% and 50%	1.02286	0.60826	1.71918	0.53287
Growth ≥ 50%	1.44608	0.85590	2.44189	0.91592
Reference person younger than 35 years old				
Financial wealth [Variation between −10% and 10%]				
Decrease ≥ 40%	0.11377	0.01164	1.10632	0.96952
Decrease between 10% and 40%	0.17570	0.04312	0.71188	0.99261
Growth between 10% and 50%	2.07079	1.03242	886.720	0.99982
Growth ≥ 50%	4.13494	0.82060	21.0620	0.93725

Adjusted by total debt, real income, sex, age, level of education, occupation, marital status, number of family members, number of family members who work, saving rate, property ownership, proportion of real estate over total wealth, and year of the survey. Reference category between brackets.

**Table 13:** Association between the variation in total debt and good and very good self-perceived health.

	Odds Ratio	95% Credibility Interval		Prob(|Log(OR)|)>0
Reference person 75 years or older				
Total debt [Variation between −10% and 10%]				
Decrease ≥ 30%	1.37242	0.71938	2.61970	0.83259
Decrease between 10% and 30%	1.06893	0.53166	1. 89509	0.77183
Growth between 10% and 50%	0.48529	0.14080	1.67085	0.87498
Growth ≥ 50%	0.78479	0.28650	2.15157	0.68042
Reference person 65–74 years				
Total debt [Variation between −10% and 10%]				
Decrease ≥ 30%	1.48688	1.04546	2.11530	0.98654
Decrease between 10% and 30%	1.46268	0.81393	2.62979	0.90898
Growth between 10% and 50%	0.80233	0.43645	1.47419	0.76213
Growth ≥ 50%	0.68780	0.42682	1.10791	0.93861
Reference person 55–64 years				
Total debt [Variation between −10% and 10%]				
Decrease ≥ 30%	1.95128	1.10503	3.44724	0.98954
Decrease between 10% and 30%	1.50496	1.09098	1.95128	0.99373
Growth between 10% and 50%	0.63200	0.41860	0.95447	0.98563
Growth ≥ 50%	0.54288	0.34978	0.84228	0.99687

Adjusted by wealth, real income, sex, age, level of education, occupation, marital status, number of family members, number of family members who work, saving rate, property ownership, proportion of real estate over total wealth, and year of the survey. Reference category between brackets.

**Table 14:** Association between the variation in total debt and good and very good self-perceived health.

	Odds Ratio	95% Credibility Interval		Prob (|log(OR)|) > 0
Reference person 45–54 years				
Total debt [Variation between −10% and 10%]				
Decrease ≥ 30%	1.65334	1.05849	2.58344	0.98662
Decrease between 10% and 30%	1.17776	0.84753	1.63710	0.83608
Growth between 10% and 50%	0.82520	0.57272	1.18860	0.84987
Growth ≥ 50%	0.57548	0.40723	0.81300	0.99916
Reference person 35–44 years				
Total debt [Variation between −10% and 10%]				
Decrease ≥ 30%	1.80905	0.84773	3.86288	0.91947
Decrease between 10% and 30%	1.45697	0.85933	2.47129	0.93789
Growth between 10% and 50%	0.74800	0.41683	1.34144	0.83598
Growth ≥ 50%	0.53216	0.31627	0.89503	0.99142
Reference person younger than 35 years old				
Total debt [Variation between −10% and 10%]				
Decrease ≥ 30%	8.65551	0.00011	6.7387 × 10^5^	0.64547
Decrease between 10% and 30%	1.45177	0.23805	8.86135	0.65604
Growth between 10% and 50%	0.33678	0.06036	1.87750	0.90385
Growth ≥ 50%	0.24909	0.03324	1.86463	0.91195

Adjusted by wealth, real income, sex, age, level of education, occupation, marital status, number of family members, number of family members who work, saving rate, property ownership, proportion of real estate over total wealth, and year of the survey. Reference category between brackets.

**Table 15:** Association between the variation in real income and good and very good self-perceived health.

	Odds Ratio	95% Credibility Interval		Prob (|log(OR)|) > 0
Reference person 75 years or older				
Real income [Variation between −10% and 10%]				
Decrease ≥ 40%	1.00092	0.75857	1.32040	0.50144
Decrease between 10% and 40%	1.06174	0.81513	1.38267	0.67080
Growth between 10% and 50%	1.49527	1.13193	1.97478	0.99771
Growth ≥ 50%	2.23405	1.67579	2.97757	1.00000
Reference person 65–74 years				
Real income [Variation between −10% and 10%]				
Decrease ≥ 40%	0.64451	0.50768	0.81804	0.99985
Decrease between 10% and 40%	0.91343	0.71456	1.16741	0.76639
Growth between 10% and 50%	1.00842	0.79086	1.28557	0.52583
Growth ≥ 50%	1.77288	1.39978	2.24496	0.99999
Reference person 55–64 years				
Real income [Variation between −10% and 10%]				
Decrease ≥ 40%	0.76086	0.56933	1.01656	0.94813
Decrease between 10% and 40%	0.90132	0.66171	1.22738	0.74634
Growth between 10% and 50%	1.13132	0.84686	1.51096	0.79775
Growth ≥ 50%	1.57959	1.19406	2.089105	0.99933

Adjusted by wealth, total debt, sex, age, level of education, occupation, marital status, number of family members, number of family members who work, saving rate, property ownership, proportion of real estate over total wealth, and year of the survey. Reference category between brackets.

**Table 16:** Association between the variation in real income and good and very good self-perceived health.

	Odds Ratio	95% Credibility Interval		Prob (|log(OR)|) > 0
Reference person 45–54 years				
Real income [Variation between −10% and 10%]				
Decrease ≥ 40%	0.81498	0.60667	1.09453	0.91371
Decrease between 10% and 40%	0.83223	0.62368	1.11023	0.81490
Growth between 10% and 50%	1.04723	0.76799	1.42762	0.61381
Growth ≥ 50%	1.14971	0.85250	1.55014	0.81937
Reference person 35–44 years				
Real income [Variation between −10% and 10%]				
Decrease ≥ 40%	0.60664	0.36722	1.00176	0.97492
Decrease between 10% and 40%	0.68319	0.41000	1.13788	0.92897
Growth between 10% and 50%	1.00537	0.59659	1.69354	0.50689
Growth ≥ 50%	1.33690	0.74547	2.39908	0.83598
Reference person younger than 35 years old				
Decrease ≥ 40%	0.08907	0.01629	0.47712	0.99771
Decrease between 10% and 40%	0.18628	0.03939	0.86980	0.98398
Growth between 10% and 50%	1.60339	0.38287	6.77327	0.73932
Growth ≥ 50%	1.83559	0.28857	11.765	0.74099

Adjusted by wealth, total debt, sex, age, level of education, occupation, marital status, number of family members, number of family members who work, saving rate, property ownership, proportion of real estate over total wealth, and year of the survey. Reference category between brackets.
